# Potential Risks to Stable Long-term Outcome of Allogeneic Hematopoietic Stem Cell Transplantation for Children With Cerebral X-linked Adrenoleukodystrophy

**DOI:** 10.1001/jamanetworkopen.2018.0769

**Published:** 2018-07-20

**Authors:** Jörn-Sven Kühl, Jana Kupper, Hermann Baqué, Wolfram Ebell, Jutta Gärtner, Christoph Korenke, Birgit Spors, Ingo G. Steffen, Gabriele Strauss, Sebastian Voigt, Bernhard Weschke, Almuth Weddige, Wolfgang Köhler, Robert Steinfeld

**Affiliations:** 1 Department of Pediatric Hematology/Oncology/Hemostaseology, University Hospital Leipzig, Leipzig, Germany; 2 Department of Pediatric Oncology/Hematology/SCT, Charité Campus Virchow-Klinikum, Berlin, Germany; 3 Department of Pediatric Neurology, Charité Campus Virchow-Klinikum, Berlin, Germany; 4 Department of Pediatric Neurology, University Medical Center Göttingen, Göttingen, Germany; 5 Department of Pediatric Neurology, Klinikum Oldenburg, Oldenburg, Germany; 6 Department of Pediatric Radiology, Charité Campus Virchow-Klinikum, Berlin, Germany; 7 Department of Pediatrics, Helios-Klinikum Berlin-Buch, Berlin, Germany; 8 Department of Neurology, University Hospital Leipzig, Leipzig, Germany

## Abstract

**Importance:**

Allogeneic hematopoietic stem cell transplantation is the standard intervention for childhood cerebral X-linked adrenoleukodystrophy. However, the pretransplant conditions, demyelination patterns, complications, and neurological outcomes of this therapy are not well characterized.

**Objectives:**

To identify the risks to stable neurocognitive survival after hematopoietic stem cell transplantation and to describe subgroups of patients with distinct clinical long-term outcomes.

**Design, Setting, and Participants:**

This case series analyzed the treatment and outcome of a cohort of 36 boys who underwent hematopoietic stem cell transplantation at Charité Universitätsmedizin Berlin, Germany, between January 1, 1997, and October 31, 2014. Case analysis was performed from January 1, 2016, through November 30, 2017. During this retrospective review, the adrenoleukodystrophy-disability rating score and the neurological function score were used. Demyelinating lesions in the brain were quantified by the Loes score.

**Main Outcomes and Measures:**

Overall survival, survival without major functional disabilities, and event-free survival were analyzed. Patients’ clinical symptoms, demyelination patterns, and stem cell source were stratified.

**Results:**

Of the 36 boys who underwent hematopoietic stem cell transplantation, the median (range) age was 7.2 (4.2-15.4) years; 18 were presymptomatic and 18 were symptomatic. Twenty-seven patients (75%) were alive at a median (interquartile range [IQR]) follow-up of 108 (40-157) months. Sixteen of 18 presymptomatic patients (89%) survived, and 13 (72%) had an event-free survival with a median (IQR) survival time of 49 (37-115) months. Among the symptomatic patients, 11 of 18 (61%) survived, but only 1 was an event-free survival (6%) (median [IQR] time, 9 [3-22] months). Of the 9 patients who received a bone marrow transplant from a matched family donor, all survived. Among the 36 patients, 6 disease-related deaths (17%) and 3 transplant-related deaths (8%) occurred. Deaths from disease progression (n = 6) occurred only in patients with demyelination patterns other than parieto-occipital. In total, 18 patients (50%) displayed limited parieto-occipital (Loes score <9) or frontal (Loes score <4) demyelination before transplant (favorable). None of these patients died of progressive disease or developed major functional disabilities, 15 of them were characterized by stable neuroimaging after the transplant, and event-free survival was 77% (95% CI, 60%-100%). In contrast, the other 18 patients with more extended parieto-occipital demyelination (n = 6), frontal involvement (n = 4), or other demyelination patterns (n = 8) progressed (unfavorable): 13 patients developed epilepsy and 10 developed major functional disabilities, and their event-free survival was 0%. This newly defined neuroimaging assessment correlated best with neurocognitive deterioration after transplant (hazard ratio, 16.7; 95% CI, 4.7-59.6).

**Conclusions and Relevance:**

All patients with favorable neuroimaging who received matched bone marrow remained stable after transplant, while some of the other patients developed major functional disabilities. Newborn screening for the disease and regular neuroimaging are recommended, and patients who lack a matched bone marrow donor may need to find new therapeutic options.

## Key Points

**Question:**

What are the risks to stable neurocognitive outcome after allogeneic hematopoietic stem cell transplantation for childhood cerebral X-linked adrenoleukodystrophy?

**Findings:**

This single-center case series involving 36 boys with cerebral X-linked adrenoleukodystrophy found that all patients with favorable neuroimaging and matched bone marrow transplant had stable neurocognitive survival. In contrast, the disease progressed for all patients without these factors, and many developed major functional disabilities.

**Meaning:**

Patients without matched bone marrow transplant or with unfavorable neuroimaging findings may need therapeutic options other than hematopoietic stem cell transplantation.

## Teaser

This case series examines the factors that contribute to disease progression and neurological deterioration in children who undergo hematopoietic stem cell transplantation to treat cerebral X-linked adrenoleukodystrophy.

## Introduction

X-linked adrenoleukodystrophy (X-ALD) is a peroxisomal disorder with an estimated incidence of 1 in 20 000 live births. It is associated with mutations in the *ABCD1* gene (OMIM 300100) that lead to defective β-oxidation with a characteristic accumulation of very long–chain fatty acids.^1,2,3,4^

In childhood, 30% to 35% of all affected males will develop an acute inflammatory cerebral variant termed *childhood cerebral X-ALD* (CCALD). This disease leads to rapid white matter destruction as well as loss of cognitive and neurological functions that usually result in death within a few years after onset of symptoms. A lack of both genotype/phenotype correlation and validated biomarkers hampers the early diagnosis of CCALD. Instead, regular magnetic resonance imaging (MRI) of the brain in affected patients is needed to diagnose CCALD as early as possible.^2^ Independent from cerebral demyelination, patients may develop primary Addison disease at any time.

Allogeneic hematopoietic stem cell transplantation (HSCT) is an established long-term treatment method for boys with CCALD.^5,6,7,8,9,10,11,12,13^ The mechanism of action seems to rely on the replacement of defective microglia by bone marrow–derived long-lived macrophages of the allogeneic donor,^14,15,16^ which is facilitated by using busulfan in the chemoconditioning regimen.^17^ Recently, lentivirus-based gene therapy has been introduced as the new treatment option for CCALD.^18,19^ Both HSCT^5,7,11^ and gene therapy^19^ are effective when performed early in the course of CCALD, when limited brain demyelination has occurred and in the absence of neurological deficits. Consequently, expanding X-ALD screening to newborns allows early diagnosis and treatment.^20^ Despite the potentials of gene therapy, HSCT will probably remain a standard in the near future. Therefore, we analyzed a single-center series of 36 patients with CCALD treated with HSCT from matched donors after a uniform myeloablative chemoconditioning. Our objectives were to identify the potential risk factors in stable neurocognitive survival after HSCT and to describe subgroups of patients with distinct clinical long-term outcomes.

## Methods

This study was approved by the institutional review board of Charité Universitätsmedizin Berlin, Germany, and was performed according to the Declaration of Helsinki.^21^ Custodial parents and patients, when appropriate, signed an institutional review board–approved informed consent form before HSCT. Patient consent for this study was waived by the head of the Ethikkomission Charité (institutional review board) as no patient was contacted and all information was taken from medical records only. This study followed the reporting guideline for case series.

### Patients

Thirty-six boys underwent HSCT for CCALD at the Charité Universitätsmedizin Berlin, Germany, between January 1, 1997, and October 31, 2014. Their transplant course and outcome, according to medical records, were retrospectively analyzed in an explorative fashion until November 30, 2017. The first 6 patients, treated before 2001, were analyzed in a previous multicenter study.^8^ Case analysis was performed from January 1, 2016, through November 30, 2017.

Diagnosis of X-ALD was based on elevated concentrations of very long–chain fatty acids in fasting plasma. In 22 patients, *ABCD1* gene (HGNC 61) mutations were also available. Gadolinium enhancement in cerebral demyelinating lesions was required for diagnosing CCALD and detected by MRI in 35 patients. One patient (patient 26) displayed a progressive brainstem lesion without contrast enhancement.

Patients underwent detailed extended neurological examinations, MRI scans, and neuropsychological testing before and after HSCT. The treatment was offered on an individually selected compassionate basis in accordance with the practice guidelines of the Working Party on Inborn Errors of the European Bone Marrow Transplant Group.^22^

### Transplants

Nine patients (25%) received bone marrow from a 10 of 10 human leukocyte antigen (HLA)–matched family donor (sibling: n = 8; father: n = 1) (Table 1). Twenty-seven patients (75%) received stem cells from 6 of 6 HLA–matched (HLA-A, -B, -DRB1) unrelated donors on the basis of low-resolution class I and high-resolution class II DNA typing: 17 received bone marrow, 9 received peripheral blood stem cells, and 1 received cord blood. High-resolution HLA class I (HLA-A, B, Cw) and class II (HLA-DRB1, DQB1) molecular typing was available on 22 unrelated transplants, of which 20 patients were HLA matched by at least 9 of 10. Myeloablative conditioning consisted of busulfan (4 mg/kg/d orally for 4 days, together with anticonvulsive prophylaxis) and cyclophosphamide (50 mg/kg/d intravenously for 4 days or 60 mg/kg/d intravenously for 2 days [n = 6; since 2012]). For prophylaxis of graft-vs-host disease (GVHD), patients received serotherapy (except patient 7) and cyclosporine intravenously. Serotherapy consisted of horse antithymocyte globulin (n = 1; 1997), rabbit antithymocyte globulin (from Genzyme; n = 10; 1998-2001 or from Fresenius [now Neovii]; n = 22; 2001-2013), and alemtuzumab (Genzyme) (n = 2; 2014). Additional GVHD prophylaxis varied (eFigure 1 in the Supplement).

**Table 1.** Patient Demographics and Transplant Characteristics

Variable	No. (%)
All patients	36 (100)
Reason for diagnosis	
Family screening	19 (53)
Addison disease	8 (22)
Behavioral or neurological symptoms	9 (25)
Addison disease	
Clinical signs	17 (47)
Clinical symptoms at HSCT	
Presymptomatic^a^	18 (50)
Clinical symptom scores at HSCT	
ALD-DRS	
0 / 1 />1^b^	20 (56) / 9 (25) / 7 (19)
Median (range)	0 (0-3)
NFS	
0 / 1 />1^b^	21 (58) / 8 (22) / 7 (19)
Median (range)	0 (0-8)
IQ before HSCT	
Performance IQ <80	12 (33)
Overall IQ, median (range [IQR])	89 (68-128 [83-103])
Neuroimaging before HSCT	
Follow-up MRI twice per year	15 (42)
Cerebral disease on first MRI	17 (47)
Loes score <9^b^	28 (78)
Loes score, median (range)	4.5 (1-14)
Interval CCALD–HSCT, median (range [IQR]), mo^c^	3 (1-30 [2-4])
Age at HSCT, median (range [IQR]), y	7.2 (4.2-15.4 [6.5-9.7])
Chemoconditioning regimen	
Busulfan and cyclophosphamide, 200 mg/kg	30 (83)
Busulfan and cyclophosphamide, 120 mg/kg	6 (17)
Donor type	
Related bone marrow^d^	9 (25)
Unrelated bone marrow	17 (47)
Unrelated peripheral blood stem cells	9 (25)
Unrelated cord blood	1 (3)
HLA match	
10 of 10 HLA high-resolution typing	22 (61)
9 of 10 HLA high-resolution typing	7 (19)
6 of 6 HLA low-/high-resolution typing	7 (19)

Abbreviations: ALD-DRS, adrenoleukodystrophy disability rating score; CCALD, childhood cerebral X-linked adrenoleukodystrophy; HLA, human leukocyte antigen; HSCT, hematopoietic stem cell transplantation; IQR, interquartile range; MRI, magnetic resonance imaging; NFS, neurological function score.

^a^ Presymptomatic: no neurological, psychological, or behavioral symptoms in detailed examination before HSCT (ie, NFS = 0; ALD-DRS = 0).

^b^ ALD-DRS range, 0-4 points; NFS range, 0-25 points; and Loes MRI score range, 0-34 points. For all scores: 0 indicates no abnormalities, and higher numbers indicate worsening status.

^c^ Interval from first detection of CCALD in MRI to HSCT.

^d^ Related donors: 6 male donors tested by very long–chain fatty acids (all negative/wild-type); 3 female donors tested by molecular genetics (*ABCD1* gene)—wild-type (n = 2) and heterozygous (n = 1).

*Engraftment* was defined as the first day of neutrophil count greater than 500/μL for 3 consecutive days. Donor chimerism was determined by short tandem repeat analysis on total nucleated cells. Diagnosis of GVHD was primarily based on clinical criteria. Staging of acute and chronic GVHD was done according to published criteria^23,24^; chronic GVHD was only differentiated into limited or extended form. Transplant toxicity was described according to the National Cancer Institute common terminology criteria for adverse events, with severe toxicity recorded for adverse events greater than grade 2. Follow-up time was calculated in surviving patients.

### Data Acquisition and Assessment of Outcome

Patient-related clinical information was obtained from a retrospective review of medical records. The following assessment tools were used: the ALD-disability rating score (ALD-DRS; range, 0-4 points) for requirements of assistance in daily life, especially in school,^5^ and the neurological function score (NFS; range, 0-25 points) for neurological deficits,^7^ including major functional disability (MFD).^19^ Demyelinating lesions in the brain were quantified by the Loes MRI severity score (range, 0-34 points).^25^ For all of these tools, a score of 0 indicates no abnormalities, whereas increasing numbers indicate worsening patient status. Patterns of MRI abnormalities were subdivided into 5 groups as described elsewhere^26^ with 1 modification (eAppendix in the Supplement). The overall IQ as well as verbal and performance IQ were measured with appropriate tools.^8^

Patients’ baseline status was analyzed according to the reason for diagnosis (ie, family screening vs Addison disease vs symptoms), the clinical disease stage, and MRI demyelination. Major outcome measures were overall survival, MFD-free survival, and event-free survival (EFS)—ie, survival without gain in ALD-DRS (eAppendix in the Supplement). For most patients, the neurological scores reflect their status 5 years after HSCT; if this time point was not reached, then the status at last visit (minimum of 37 months after HSCT) or just before death was used.

### Statistical Analysis

Comparisons of continuous variables were done with the Mann-Whitney rank sum test. Categorical variables were compared using the Fisher exact test. Survival was estimated by the Kaplan-Meier method, and comparisons were done with the log-rank method. The Cox proportional hazards regression model was used to identify risk factors on overall survival, MFD-free survival, and EFS. Statistical analyses were performed using Sigmaplot, version 11.0 (Systat Inc) and R, version 3.4.3 (R Foundation for Statistical Computing). Two-sided *P* < .05 was considered as significant.

## Results

### Patient Status Before HSCT

Patient characteristics are summarized in Table 1. The median (range) age of the 36 boys was 7.2 (4.2-15.4) years. Thirteen of 18 presymptomatic patients (72%) (ALD-DRS and NFS = 0) were diagnosed by family screening, and 17 of 36 patients (47%) displayed clinical symptoms of adrenal insufficiency. Early diagnosis by family screening or Addison disease allowed for MRI scans every 6 months in 15 patients (42%) before detection of CCALD.

Among the 18 symptomatic patients, 9 (50%) were admitted for behavioral problems. One of these patients was initially diagnosed by family screening but did not present for regular MRI examinations. Another patient had afebrile seizures without apparent school problems at diagnosis. Eight patients (44%) revealed discrete neurological or cognitive abnormalities at examination prior to HSCT. Two of these 8 patients were diagnosed with Addison disease, 2 had missed regular neuroimaging scans, and in 4 patients HSCT was delayed due to rare MRI patterns. The 18 symptomatic patients, with a median (interquartile range [IQR]) age of 9.4 (8.3-11.6) years, were older than the presymptomatic patients (median [IQR] age, 6.7 [5.7-7.0] years) and 14 of 18 underwent HSCT before 2004.

### Survival, Transplant Characteristics, Toxic Effects, and GVHD

Twenty-seven patients (75%) were alive at a median (IQR) follow-up of 108 (40-157) months. The overall 5-year survival rate was 81 % (95% CI, 69%-95%). After receiving a bone marrow transplant from a matched family donor, all 9 patients survived. The estimated 10-year survival after a matched unrelated donor transplant was 68% (95% CI, 52%-90%; log-rank, 3.81; *P* = .051). Among the 36 patients, 6 disease-related deaths (17%) and 3 transplant-related deaths (8%) occurred. Two patients died of complications after acute GVHD grade 4: one had received bone marrow and the other peripheral blood stem cells, both from 10 of 10 HLA–matched donors with complete GVHD prophylaxis (rabbit antithymocyte globulin [Fresenius], cyclosporine, and mycophenolate mofetil). The third boy died from late adenovirus reactivation after a cord blood transplant. Death from CCALD progression occurred at a median (range) time of 29 (4-185) months. Two severely progressed, bedridden patients died of pneumonia, 1 at 100 months and another at 185 months after HSCT.

The median (range) time to donor engraftment (absolute neutrophil count >500/μL) was 15 (10-32) days after bone marrow transplant and 12 (11-16) days after peripheral blood stem cell transplant (*P* = .03). No graft failures were encountered. Stable donor chimerism (>95%) was observed in 31 of 35 evaluable patients (89%); mixed chimeras were detected only after serotherapy with horse antithymocyte globulin (Genzyme). Hemorrhagic cystitis (n = 24) and severe infections greater than grade 2 (n = 14) were the most common toxic effects and tended to occur more often in symptomatic patients. Severe central nervous system toxic effects were noticed: seizures with use of busulfan (n = 2), posterior reversible encephalopathy syndrome (n = 1), and central nervous system hemorrhage (n = 1) associated with acute GVHD grade 4. Acute GVHD grade 2 or greater was observed in 9 patients (25%), and extensive chronic GVHD was observed in 8 patients (22%), which was steroid responsive and resolved in 6 of them.

### Outcome According to Clinical Baseline Status

Sixteen of 18 presymptomatic patients (89%) survived, and 13 (72%) had an EFS with a median (IQR) survival time of 49 (37-115) months. Among the symptomatic patients, 11 of 18 (61%) survived, but only 1 was EFS (6%) (median [IQR] EFS time, 9 [3-22] months). Death from X-ALD progression (n = 6), development of MFD (n = 10), and epilepsy (n = 13) occurred only in symptomatic patients, especially in those 10 patients who were admitted because of behavioral or neurological symptoms: 4 died, 8 developed MFD, and 9 had epilepsy. Overall IQ before HSCT did not differ between symptomatic (median [range] IQ, 89 [68-114]) and presymptomatic (median [range] IQ, 94 [77-128]) patients. However, there was a trend of a higher proportion of symptomatic patients with a performance IQ lower than 80 (8 of 18 vs 4 of 18 patients).

Twenty patients (56%) had a normal baseline ALD-DRS, of whom 13 (65%) remained stable. Only 1 of 16 patients (6%), with a baseline ALD-DRS greater than 0 and his brother as the donor, had minimal difficulties at school and maintained his disability level. Of the 7 patients who were progressing despite a normal baseline ALD-DRS, 4 developed GVHD or chronic infection after non–bone marrow transplants, and 3 patients displayed rare demyelination patterns. Eighteen of 21 patients (86%) without previous neurological deficits (NFS = 0) had an MFD-free survival, 17 (81%) did not develop deficits after a transplant (change in NFS = 0), and 15 (71%) had an EFS. All 15 patients with a baseline NFS greater than 0 deteriorated neurologically after HSCT.

Estimates for times and rates of overall survival, MFD-free survival, and EFS of the entire cohort as well as hazard ratios for different covariates are presented in Table 2 and eFigure 2 in the Supplement. Despite the late deaths among progressed patients, no MFD or progression in ALD-DRS developed later than 38 months after the transplant. In addition to advanced clinical baseline status, transplant complications such as an infection greater than grade 2, acute GVHD grade 2 or greater, and extensive chronic GVHD had an association with negative outcome in a univariable analysis. Furthermore, stable patients (change in ALD-DRS = 0) had an earlier immune reconstitution with more naive T-helper cells (median [range], 17/μL [1-183/μL]; n = 12) at 6 months after HSCT than patients who deteriorated (median [range], 2.5/μL [0-53/μL]; n = 20; *P* < .05).

**Table 2.** Analysis of Transplant Outcomes

Variable	No. (%)	Survival		
		Overall	MFD-Free^a^	Event-Free^a^
All patients [events]	36 (100)	[8]	[13]	[22]
**Kaplan-Meier Estimates**^b^				
Survival times, mo				
Median (IQR)	36 (100)	108 (40-156)	69 (23-131)	26 (9-60)
10-y Survival probability, %				
Mean (95% CI)	36 (100)	77 (64-92)	64 (50-82)	39 (26-59)
**Hazard Ratios (95% CI)**^c^				
Neurological status at HSCT				
Presymptomatic	18 (50)	0.31 (0.06-1.5)	0.13 (0.03-0.57)	0.12 (0.04-0.33)
NFS ≤1^d^	29 (81)	0.29 (0.07-1.2)	0.11 (0.04-0.34)	0.11 (0.04-0.31)
ALD-DRS = 0^d^	20 (56)	0.43 (0.10-1.8)	0.15 (0.04-0.56)	0.17 (0.07-0.42)
Neuroimaging before HSCT				
Loes score ≥9^d^	8 (22)	0.48 (0.06-4.0)	3.0 (1.0-9.1)	4.4 (1.8-10.9)
Cerebellar pattern^e^	4 (11)	12.1 (3.0-49.0)	6.5 (2.0-21.5)	6.2 (1.9-20.9)
Projection fibers involved	21 (58)	1.3 (0.3-5.3)	2.1 (0.6-6.7)	4.0 (1.5-11.1)
Unfavorable MRI	18 (50)	3.2 (0.7-16.1)	8.0 (1.8-36.1)	16.7 (4.7-59.6)
Transplant characteristics				
Related donor	9 (25)	NA^f^	0.20 (0.03-1.5)	0.50 (0.17-1.5)
Bone marrow	26 (72)	0.67 (0.16-2.8)	0.93 (0.29-3.0)	0.84 (0.34-2.1)
Engraftment before day 14 after HSCT	16 (44)	1.2 (0.31-5.0)	1.2 (0.39-3.5)	1.4 (0.62-3.3)
Infection grade >2	14 (39)	5.5 (1.1-27.4)	7.0 (1.9-25.9)	3.0 (1.3-7.0)
Acute GVHD grade ≥2	9 (25)	6.0 (1.4-25.3)	3.2 (1.1-9.7)	3.0 (1.3-7.1)
Extensive chronic GVHD	8 (22)	7.4 (1.8-31.2)	4.1 (1.3-12.3)	3.3 (1.3-8.5)

Abbreviations: ALD-DRS, adrenoleukodystrophy disability rating score; GVHD, graft-vs-host disease; HSCT, hematopoietic stem cell transplantation; IQR, interquartile range; MFD, major functional disability; MRI, magnetic resonance imaging; NA, not applicable; NFS, neurological function score.

^a^ MFD-free is survival without major functional disabilities. Event-free is survival without gain in ALD-DRS.

^b^ To allow calculation of 95% CIs, maximum follow-up was limited to 180 months after HSCT (n = 4).

^c^ Cox proportional hazards regression of covariates was used for respective outcomes.

^d^ ALD-DRS range, 0-4 points; NFS range, 0-25 points (NFS ≤1: score with maximum 1 deficit); and Loes in this MRI score range, 0-34 points. For all scores: 0 indicates no abnormalities, and higher numbers indicate worsening status.

^e^ Cerebellar pattern refers to demyelinating lesions in the cerebellum and/or basal ganglia.

^f^ Hazard ratio and 95% CI cannot be calculated due to 100% survival after related bone marrow transplants.

### Outcome Based on Neuroimaging Before HSCT

Detailed MRI characteristics of the cohort before HSCT and outcome are summarized in Table 3. Typical parieto-occipital demyelinating lesions were detected in 22 patients (61%), while 14 patients (39%) showed other demyelination patterns: frontal involvement in 6 patients (17%), and even other patterns in 8 patients (22%). In total, 18 patients (50%) displayed limited parieto-occipital (Loes score <9) or frontal (Loes score <4) demyelination before transplant (favorable MRI) and only 3 of them (17%) had a Loes score of 4.5 or greater. None of these patients died of progressive disease or developed MFDs, 15 of them were characterized by stable MRI scans after HSCT, and EFS was 77% (95% CI, 60%-100%). In contrast, all other 18 patients (50%) with more extended parieto-occipital demyelination (n = 6), frontal involvement (n = 4), or other demyelination patterns (n = 8) progressed (unfavorable MRI): 13 patients developed epilepsy and 10 developed MFDs, and their EFS was 0%. A so-defined neuroimaging assessment correlated best with neurocognitive deterioration after transplant (hazard ratio, 16.7; 95% CI, 4.7-59.6). Moreover, 5 of 6 patients with an unfavorable MRI, who died from disease progression, had a baseline Loes score of less than 9. The only 2 patients who displayed gadolinium uptake for more than 6 months after HSCT showed demyelinating lesions in the cerebellum or basal ganglia previously (eFigure 3 in the Supplement). Changes in Loes score, depending on different demyelination patterns, are illustrated in eFigure 4 in the Supplement.

**Table 3.** Brain Magnetic Resonance Imaging (MRI) Characteristics Before and After Hematopoietic Stem Cell Transplantation (HSCT)

Characteristic	MRI Characteristic (N=36)	
	Favorable, No. (LS)	Unfavorable, No. (LS)
**Status Before HSCT**		
MRI pattern^a^		
Parieto-occipital	16 (LS <9)	6 (LS ≥9)
Frontal involvement	2 (LS <4)	4 (LS ≥4)
Others (n = 8)		
Projection fibers only	NA	2 (LS: 1, 5.5)^b^^,^^c^
Cerebellum or basal ganglia	NA	4 (LS: 2.5, 8.5, 8.5, 12.5)^b^
Parieto-occipital + frontal involvement	NA	2 (LS: 8, 10)^b^
Projection fibers involved, No. (%)		
Pons	3 (17)	12 (67)^d^
Capsula interna, partial/complete^e^	5 (28) / 0	7 (39) / 5 (28)
ALD-DRS = 0	17 (94)	1 (6)^d^
NFS = 0	17 (94)	4 (22)
**Status After HSCT**		
Gadolinium enhancement >6 mo after HSCT, No. (%)^f^	0 of 16	2 of 15 (13)
Stable MRI during first year after HSCT^g^	15 (83)	4 (22)^d^
Epilepsy	0	13 (72)^d^
**Change in LS 12 mo After HSCT**		
Median (range [IQR])	1.0 (0 to 2.0 [0 to 2.0])	5 (-2.5 to 10.0 [2.5 to 7.0])^d^

Abbreviations: ALD-DRS, adrenoleukodystrophy disability rating score; IQR, interquartile range; LS, Loes score; NA, not applicable; NFS, neurological function score.

^a^ Demyelination patterns according to Loes et al^25^ with modifications. Greater LS numbers indicate more demyelinating lesions.

^b^ Loes score of respective patients.

^c^ Patient 26 with demyelination of brainstem only without gadolinium enhancement.

^d^ Significance level (*P* < .05) between favorable and unfavorable for a respective characteristic.

^e^ Capsula interna partial is the incomplete or unilateral demyelination of tracts in the internal capsule.

^f^ All patients with transplant-related mortality and those who died within 12 months of disease progression were omitted (n = 5).

^g^ Change in LS of at least 2 points in the first year after HSCT.

Both MRI demyelination pattern and stem cell source allowed for the division of patients into 4 groups (Figure and Table 4). Patients with favorable MRI patterns who had a matched bone marrow donor (group Ia; n = 11) had an EFS of 100%, whereas patients who received peripheral blood stem cells or cord blood (group Ib; n = 7) had an overall survival and MFD-free survival of 71% (95% CI, 45%-100%) as well as an EFS of 43% (95% CI, 18%-100%). Among the patients with unfavorable neuroimaging, all 4 with demyelination of the cerebellum or basal ganglia died with MFD (group IIa). The other 14 patients with unfavorable MRI (group IIb) had an MFD-free survival of 50% (95% CI, 30%-84%) and an EFS of 0%.

**Figure.**
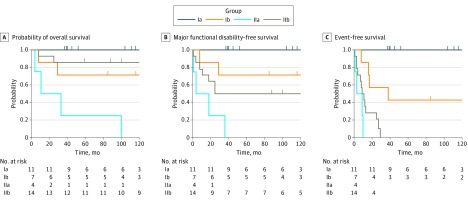
Probability of Survival for Patients with Childhood Cerebral X-linked Adrenoleukodystrophy Patients were stratified by neuroimaging before transplant and stem cell source. Group Ia (n = 11) comprised patients with favorable magnetic resonance imaging (MRI) and bone marrow donor; group Ib (n = 7), favorable MRI and other stem cell source; group IIa (n = 4), demyelination of cerebellum or basal ganglia; and group IIb (n = 14), other unfavorable MRI. Marks indicate censored patients.

**Table 4.** Patient Characteristics and Outcome by Different Subgroups

Variable	All Patients, No. (%) (N = 36)	Patients, No.			
		Favorable Neuroimaging		Unfavorable Neuroimaging	
		Bone Marrow (n = 11), Group Ia	Peripheral Stem Cells or Cord Blood (n = 7), Group Ib	With Demyelination of Cerebellum or Basal Ganglia (n = 4), Group IIa	Other MRI (n = 14), Group IIb
Neurological status at HSCT					
Presymptomatic	18 (50)	10	7	0	1
NFS ≤1^a^	29 (81)	11	7	2	9
ALD-DRS = 0^a^	20 (56)	10	7	1	2
Neuroimaging before HSCT					
Loes score ≥9^a^	8 (22)	0	0	1	7
Parieto-occipital pattern	22 (61)	9	7	0	6
Projection fibers involved	21 (58)	4	1	3	13
Transplant characteristics					
Transplant before 2004	21 (58)	4	4	4	9
Related donor	9 (25)	5	0	0	4
Bone marrow	26 (72)	11	0	3	12
Engraftment before day 14 after HSCT	16 (44)	4	4	1	7
Donor chimerism >95%	31 (89)^b^	9^b^	7	3	12
Infection grade >2	14 (39)	2	3	2	7
Acute GVHD grade ≥2	9 (25)	0	2	2	5
Outcome					
Transplant-related deaths	3 (8)	0	2	0	1
Disease-related deaths	6 (17)	0	0	4	2
Major functional disabilities	10 (28)	0	0	4	6
Event-free survivors	14 (39)	11	3	0	0
Event-free survival time, median (IQR), mo^c^	26 (9-60)	104 (45-121)	38 (17-132)	6 (2.5-9.2)	10 (4.8-22)

Abbreviations: ALD-DRS, adrenoleukodystrophy disability rating score; GVHD, graft-vs-host disease; HSCT, hematopoietic stem cell transplantation; IQR, interquartile range; MRI, magnetic resonance imaging; NFS, neurological function score.

^a^ ALD-DRS range, 0-4 points; NFS range, 0-25 points (NFS ≤1: score with maximum 1 deficit); and Loes MRI score range, 0-34 points. For all scores: 0 indicates no abnormalities, and higher numbers indicate worsening status.

^b^ Donor chimerism: determined in only of 35 patients (1 patient from group Ia was missing).

^c^ Kaplan-Meier estimates.

## Discussion

Allogeneic HSCT is currently the standard therapeutic option for patients with CCALD, who reported 5-year survival rates of 56% to 79%.^5,7,9,12,13,27,28,29^ Comparing these studies, we realized that mismatched donors, reduced-intensity chemoconditioning, and cord blood seem to be the risk factors in inferior outcome (eTable in the Supplement). This observation may explain the rather favorable results of this large single-center transplant case series. We report a 5-year survival rate of 81%, an observed transplant-related mortality of 8%, and no graft failures after matched donor transplant using a uniform myeloablative chemoconditioning. In addition, no veno-occlusive disease occurred in this series even though busulfan was administered without therapeutic drug monitoring.

Previous studies of HSCT in CCALD focused on risk factors for survival.^5,7^ Recently, MFD-free survival was introduced to characterize patients for whom treatment would prevent progression to major functional deficits.^19^ Demanding treatment modalities, such as HSCT, should also allow for neurocognitive stability. Therefore, we also used the robust ALD-DRS^5^ as an important outcome measure. The ALD-DRS reflects the need for assistance in daily life, especially at school. An EFS, as defined, indicates a stable school performance after a transplant. Only 14% of all patients with CCALD in the first international multicenter transplant cohort did not need any assistance before HSCT,^5^ but 56% of patients in our series had a normal baseline ALD-DRS, of whom 65% did not deteriorate after the transplant. None of the stable patients developed seizures or MFDs, deteriorated in their overall IQ, or showed progressive demyelination. The disability score may not necessarily detect subtle changes in some domains of the IQ,^30^ but a normal ALD-DRS precludes a relevant impairment in daily life activities and guarantees a self-determined life.

The uniform chemoconditioning allowed the analysis of the association of transplant factors with outcome: survival tended to be inferior after unrelated transplant. Even more important, absence of GVHD and relevant infections were associated with superior MFD-free survival and EFS. In addition, stable patients showed an earlier immune reconstitution. The absence of transplant-associated alloreactions may facilitate the exchange of bone marrow–derived donor macrophages in the brain and therefore allow for improved neurocognitive outcome. This possibility might also explain the inferior EFS of patients who received stem cell sources other than bone marrow, which were associated with faster engraftment, more GVHDs, and infections.

Not unexpected was that the baseline neuroimaging status was associated with posttransplant results. A Loes score of less than 9 to 10 has already been described as a critical cutoff score and a factor in superior survival, provided patients have a parieto-occipital demyelination pattern.^5,7^ However, 14 patients (39%) in this cohort showed other demyelination patterns, a proportion that is in agreement with the proportion observed in other series,^26^ and the Paris cohort according to P. Aubourg, MD, PhD (written communication, March 2018). Disease-related deaths occurred only among patients with nonparieto-occipital patterns; 5 of those 6 patients had baseline Loes scores of less than 9. In particular, all 4 patients with demyelinating lesions in the cerebellum or basal ganglia died, indicating a reduced association with HSCT in this uncommon MRI pattern. Because of the small number of patients analyzed in this study, whether this finding reflects a specific pathomechanism remains unclear. However, both the Loes score per se and the location of the demyelinating lesion are obviously important and thus may be a factor in clinical outcome. Therefore, we introduced a differentiated MRI assessment in which a Loes score of less than 9 was considered favorable for only parieto-occipital demyelination. A similar cutoff score was lowered to a Loes score of less than 4 for frontal patterns. All other rare demyelination patterns were considered unfavorable. An unfavorable MRI, as defined, became the best single indicator for neurocognitive deterioration and poorer school performance after HSCT. Our corresponding definition of favorable MRI, closely associated with stable outcome, may contradict a recent study, which found that a baseline Loes score of 4.5 or greater was already indicative of a substantial impairment in several neurocognitive tasks after a transplant.^30^ However, in our analysis, only 3 of 18 patients (17%) with favorable MRI patterns had a Loes score of 4.5 or greater.

In this case series, patients best suited for a transplant were only identified by family screening and Addison disease. In particular, patients diagnosed as family members had a fair chance to be regularly monitored before the onset of CCALD. However, motivating these patients to undergo MRI scans every 6 months remains a challenge. Eichler et al^19^ recently reported an outstanding MFD-free survival rate of 88% after gene therapy for CCALD in a group of well-selected patients without neurological deficits. An equivalent subgroup in this series achieved an MFD-free survival rate of 86%. These data argue most convincingly in favor of introducing newborn screening to be able to offer therapeutic options in time to all at-risk patients with X-ALD.

### Limitations

This study bears some limitations in that it is a retrospective case series and, due to the small size, explorative. The chemoconditioning used is known to be neurotoxic. Therefore, newer toxicity-reduced regimens, such as busulfan and fludarabine phosphate, may have been better for patients with advanced disease. Performing transplants on symptomatic patients was primarily stopped in the past because of poor results. However, we cannot exclude the possibility that presymptomatic patients who underwent transplants recently may show improvements in therapy. Furthermore, the clinical relevance of our new MRI categorization will have to be validated in a larger series. However, the identification of distinct risk groups in this series provides a basis for future prospective multicenter trials.

## Conclusions

Gene therapy has great promise,^18,19,31^ and HSCT is likely to remain an important treatment option in the near future. This study indicates that HSCT may be of most value to patients with favorable neuroimaging and matched bone marrow donors. However, patients who receive other stem cell sources may be at substantial risk for deterioration even with favorable baseline neuroimaging. In the near future, gene therapy may be an effective treatment for these patients. Patients with rare demyelination patterns, especially those with cerebellar or basal ganglia involvement, did very poorly for yet unknown reasons and should be considered for treatment options other than HSCT.

## Notes

Supplement. **eFigure 1.** Time Course of Stem Cell Transplants **eAppendix.** Definitions Used in the Study **eFigure 2A.** Probability of 10-Year Overall Survival **eFigure 2B.** Probability of Survival Without Major Functional Disabilities **eFigure 2C.** Probability of Event-Free Survival (ie, Survival Without Gain in Disability Level) **eFigure 3.** Favorable (A) and Unfavorable (B-D) Neuroimages **eFigure 4.** Demyelinating Lesions in Neuroimaging **eTable.** Overview on Retrospective Transplant Studies and Prospective Gene Therapy Study for CCALD

## References

[1] Moser HW Adrenoleukodystrophy: phenotype, genetics, pathogenesis and therapy. Brain. 1997;120(pt 8):-. doi:10.1093/brain/120.8.1485 9278636

[2] Engelen M, Kemp S, de Visser M, X-linked adrenoleukodystrophy (X-ALD): clinical presentation and guidelines for diagnosis, follow-up and management. Orphanet J Rare Dis. 2012;7:51. doi:10.1186/1750-1172-7-51 22889154 PMC3503704

[3] Wiesinger C, Eichler FS, Berger J The genetic landscape of X-linked adrenoleukodystrophy: inheritance, mutations, modifier genes, and diagnosis. Appl Clin Genet. 2015;8:109-121.doi:10.2147/TACG.S49590 25999754 PMC4427263

[4] Turk BR, Moser AB, Fatemi A Therapeutic strategies in adrenoleukodystrophy. Wien Med Wochenschr. 2017;167(9-10):219-226. doi:10.1007/s10354-016-0534-2 28493141

[5] Peters C, Charnas LR, Tan Y, Cerebral X-linked adrenoleukodystrophy: the international hematopoietic cell transplantation experience from 1982 to 1999. Blood. 2004;104(3):881-888. doi:10.1182/blood-2003-10-3402 15073029

[6] Shapiro E, Krivit W, Lockman L, Long-term effect of bone-marrow transplantation for childhood-onset cerebral X-linked adrenoleukodystrophy. Lancet. 2000;356(9231):713-718. doi:10.1016/S0140-6736(00)02629-5 11085690

[7] Miller WP, Rothman SM, Nascene D, Outcomes after allogeneic hematopoietic cell transplantation for childhood cerebral adrenoleukodystrophy: the largest single-institution cohort report. Blood. 2011;118(7):1971-1978. doi:10.1182/blood-2011-01-329235 21586746

[8] Baumann M, Korenke GC, Weddige-Diedrichs A, Haematopoietic stem cell transplantation in 12 patients with cerebral X-linked adrenoleukodystrophy. Eur J Pediatr. 2003;162(1):6-14. doi:10.1007/s00431-002-1097-3 12486501

[9] Beam D, Poe MD, Provenzale JM, Outcomes of unrelated umbilical cord blood transplantation for X-linked adrenoleukodystrophy. Biol Blood Marrow Transplant. 2007;13(6):665-674. doi:10.1016/j.bbmt.2007.01.082 17531776

[10] Aubourg P, Blanche S, Jambaqué I, Reversal of early neurologic and neuroradiologic manifestations of X-linked adrenoleukodystrophy by bone marrow transplantation. N Engl J Med. 1990;322(26):1860-1866. doi:10.1056/NEJM199006283222607 2348839

[11] Mahmood A, Raymond GV, Dubey P, Peters C, Moser HW Survival analysis of haematopoietic cell transplantation for childhood cerebral X-linked adrenoleukodystrophy: a comparison study. Lancet Neurol. 2007;6(8):687-692. doi:10.1016/S1474-4422(07)70177-1 17618834

[12] Kato S, Yabe H, Takakura H, Hematopoietic stem cell transplantation for inborn errors of metabolism: a report from the Research Committee on Transplantation for Inborn Errors of Metabolism of the Japanese Ministry of Health, Labour and Welfare and the Working Group of the Japan Society for Hematopoietic Cell Transplantation. Pediatr Transplant. 2016;20(2):203-214. doi:10.1111/petr.12672 26806759

[13] van den Broek BTA, Page K, Paviglianiti A, Early and late outcomes after cord blood transplantation for pediatric patients with inherited leukodystrophies. Blood Adv. 2018;2(1):49-60. doi:10.1182/bloodadvances.2017010645 29344584 PMC5761624

[14] Cartier N, Lewis CA, Zhang R, Rossi FM The role of microglia in human disease: therapeutic tool or target? Acta Neuropathol. 2014;128(3):363-380. doi:10.1007/s00401-014-1330-y 25107477 PMC4131134

[15] Moser HW, Mahmood A New insights about hematopoietic stem cell transplantation in adrenoleukodystrophy. Arch Neurol. 2007;64(5):631-632. doi:10.1001/archneur.64.5.631 17502460

[16] Schönberger S, Roerig P, Schneider DT, Reifenberger G, Göbel U, Gärtner J Genotype and protein expression after bone marrow transplantation for adrenoleukodystrophy. Arch Neurol. 2007;64(5):651-657. doi:10.1001/archneur.64.5.noc60105 17353371

[17] Capotondo A, Milazzo R, Politi LS, Brain conditioning is instrumental for successful microglia reconstitution following hematopoietic stem cell transplantation. Proc Natl Acad Sci U S A. 2012;109(37):15018-15023. doi:10.1073/pnas.1205858109 22923692 PMC3443128

[18] Cartier N, Hacein-Bey-Abina S, Bartholomae CC, Hematopoietic stem cell gene therapy with a lentiviral vector in X-linked adrenoleukodystrophy. Science. 2009;326(5954):818-823. doi:10.1126/science.1171242 19892975

[19] Eichler F, Duncan C, Musolino PL, Hematopoietic stem-cell gene therapy for cerebral adrenoleukodystrophy. N Engl J Med. 2017;377(17):1630-1638. doi:10.1056/NEJMoa1700554 28976817 PMC5708849

[20] Kemper AR, Brosco J, Comeau AM, Newborn screening for X-linked adrenoleukodystrophy: evidence summary and advisory committee recommendation. Genet Med. 2017;19(1):121-126. doi:10.1038/gim.2016.68 27337030 PMC5182180

[21] World Medical Association World Medical Association Declaration of Helsinki: ethical principles for medical research involving human subjects. JAMA. 2013;310(20):2191-2194. doi:10.1001/jama.2013.281053 24141714

[22] Peters C, Steward CG; National Marrow Donor Program; International Bone Marrow Transplant Registry; Working Party on Inborn Errors, European Bone Marrow Transplant Group Hematopoietic cell transplantation for inherited metabolic diseases: an overview of outcomes and practice guidelines. Bone Marrow Transplant. 2003;31(4):229-239. doi:10.1038/sj.bmt.1703839 12621457

[23] Przepiorka D, Weisdorf D, Martin P, 1994 Consensus conference on acute GVHD grading. Bone Marrow Transplant. 1995;15(6):825-828. 7581076

[24] Shulman HM, Sullivan KM, Weiden PL, Chronic graft-versus-host syndrome in man: a long-term clinicopathologic study of 20 Seattle patients. Am J Med. 1980;69(2):204-217. doi:10.1016/0002-9343(80)90380-0 6996481

[25] Loes DJ, Hite S, Moser H, Adrenoleukodystrophy: a scoring method for brain MR observations. AJNR Am J Neuroradiol. 1994;15(9):1761-1766. 7847225 PMC8333737

[26] Loes DJ, Fatemi A, Melhem ER, Analysis of MRI patterns aids prediction of progression in X-linked adrenoleukodystrophy. Neurology. 2003;61(3):369-374. doi:10.1212/01.WNL.0000079050.91337.83 12913200

[27] Tran C, Patel J, Stacy H, Long-term outcome of patients with X-linked adrenoleukodystrophy: a retrospective cohort study. Eur J Paediatr Neurol. 2017;21(4):600-609. doi:10.1016/j.ejpn.2017.02.006 28274546

[28] Mitchell R, Nivison-Smith I, Anazodo A, Outcomes of hematopoietic stem cell transplantation in primary immunodeficiency: a report from the Australian and New Zealand Children’s Haematology Oncology Group and the Australasian Bone Marrow Transplant Recipient Registry. Biol Blood Marrow Transplant. 2013;19(3):338-343. doi:10.1016/j.bbmt.2012.11.619 23228588

[29] Fernandes JF, Bonfim C, Kerbauy FR, Haploidentical bone marrow transplantation with post transplant cyclophosphamide for patients with X-linked adrenoleukodystrophy: a suitable choice in an urgent situation. Bone Marrow Transplant. 2018;53(4):392-399. doi:10.1038/s41409-017-0015-2 29330393

[30] Pierpont EI, Eisengart JB, Shanley R, Neurocognitive trajectory of boys who received a hematopoietic stem cell transplant at an early stage of childhood cerebral adrenoleukodystrophy. JAMA Neurol. 2017;74(6):710-717. doi:10.1001/jamaneurol.2017.0013 28418523 PMC5540007

[31] Cartier N, Hacein-Bey-Abina S, Bartholomae CC, Lentiviral hematopoietic cell gene therapy for X-linked adrenoleukodystrophy. Methods Enzymol. 2012;507:187-198. doi:10.1016/B978-0-12-386509-0.00010-7 22365775

