# Supplementary material for: Potential Risks to Stable Long-term Outcome of Allogeneic Hematopoietic Stem Cell Transplantation for Children With Cerebral X-linked Adrenoleukodystrophy
Source: JAMA Cardiol. 2018 Jul 20;1(3):e180769. doi: 10.1001/jamanetworkopen.2018.0769 (PMC6324299; doi:10.1001/jamanetworkopen.2018.0769)
Supplement: Supplement. — eFigure 1. Time Course of Stem Cell Transplants eAppendix. Definitions Used in the Study eFigure 2A. Probability of 10-Year Overall Survival eFigure 2B. Probability of Survival Without Major Functional Disabilities eFigure 2C. Probability of Event-Free Survival (ie, Survival Without Gain in Disability Level) eFigure 3. Favorable (A) and Unfavorable (B-D) Neuroimages eFigure 4. Demyelinating Lesions in Neuroimaging eTable. Overview on Retrospective Transplant Studies and Prospective Gene Therapy Study for CCALD [file jamanetwopen-1-e180769-s001.pdf]

Supplementary Online Content

Kühl J-S, Kupper J, Baqué H, et al. Potential risks to stable long-term outcome of allogeneic stem cell transplantation for children with cerebral X-linked adrenoleukodystrophy. *JAMA Netw Open.* 2018;1(3):e180769. doi:10.1001/jamanetworkopen.2018.0769

eFigure 1. Time Course of Stem Cell Transplants

eAppendix. Definitions Used in the Study

eFigure 2A. Probability of 10-Year Overall Survival

eFigure 2B. Probability of Survival Without Major Functional Disabilities

eFigure 2C. Probability of Event-Free Survival (ie, Survival Without Gain in Disability Level)

eFigure 3. Favorable (A) and Unfavorable (B-D) Neuroimages

eFigure 4. Demyelinating Lesions in Neuroimaging

eTable. Overview on Retrospective Transplant Studies and Prospective Gene Therapy Study for CCALD

This supplementary material has been provided by the authors to give readers additional information about their work.

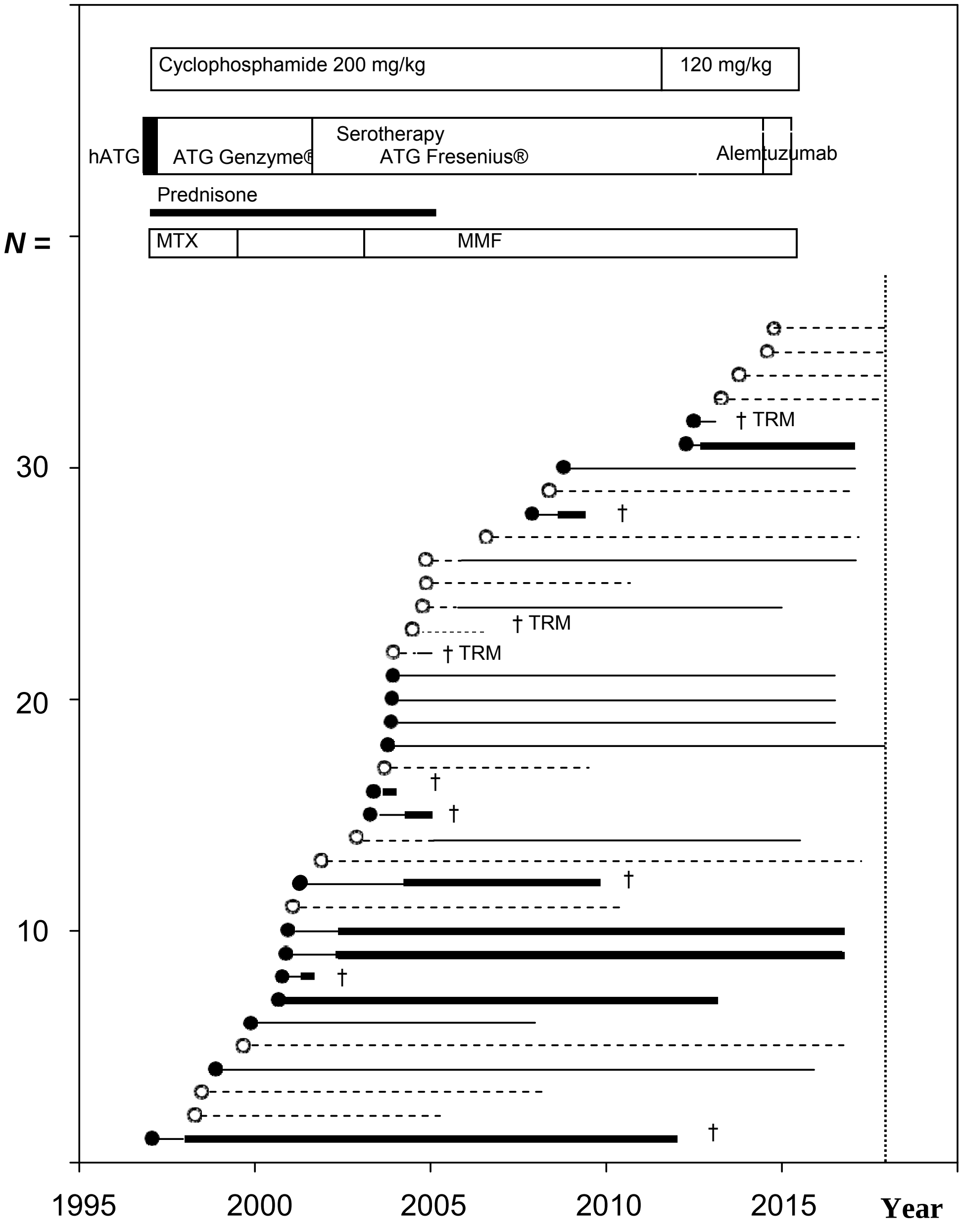

eFigure 1. Time Course of Stem Cell Transplants

Shown are the time points for transplantation with follow-up of all 36 patients. Changes in cyclophosphamide (dose reduction since 2012), serotherapy (ATG Genzyme® in patients 2-12, ATG Fresenius® in patients 13-34, alemtuzumab in patients 35-36) and other graft-versus-host prophylaxis (MTX in patients 1-6, prednisone in most patients until 2004 (*N*=18) and MMF in some patients since 2003 (*N*=8)) are indicated above.

hATG: horse anti-thymocyte globulin; MTX: methotrexate; MMF: mycophenolate mofetil; TRM: transplant-related mortality. Closed circles: symptomatic patients; open circles: pre-symptomatic patients at transplant; fine lines: symptomatic follow-up; bars: development of major functional disabilities; broken lines: pre-symptomatic follow-up; †: deceased patients. The dotted vertical line indicates end of study.

© 2018 Kühl J-S et al. *JAMA Network Open.*

eAppendix. Definitions Used in the Study

Major functional disabilities (MFD) (Eichler F et al., N Engl J Med. 2017; 377: 1630):
Status with any of the following:

- loss of communication
- cortical blindness
- tube feeding
- wheelchair bound
- no voluntary movements
- total incontinence

MFD-free survival – Survival without any MFD.

Event-free survival (EFS) – Survival without any deterioration in ALD disability rating score (ALD-DRS) [baseline ALD-DRS = 4 excluded]. Any deterioration in school performance (including repeating class or change to less demanding school) or significant deterioration in overall, verbal or performance IQ (ΔIQ > 14) was considered as change in ALD-DRS.

MRI patterns (modified from Loes DJ et al., Neurology. 2003; 61: 369):

- #1: parietooccipital (including splenium corporis callosum)
- #2: frontal (including genu corporis callosum)
- #3 long-tract fibers only
- #4 cerebellum **and all patterns with basal ganglia involvement (modification)**
- #5 parietooccipital and frontal (or splenium and genu involved)

Favorable MRI – Pattern #1 with Loes score < 9 or pattern #2 with Loes score < 4.

Unfavorable MRI – More advanced patterns 1 & 2 or all other, rare patterns.

Stable MRI post-HSCT – Gain ≤ 2 points in Loes score (ΔLoes score ≤ 2) within the first year post-transplant in the absence of Gadolinium enhancement.

Progressive MRI post-HSCT – Anything different from stable.

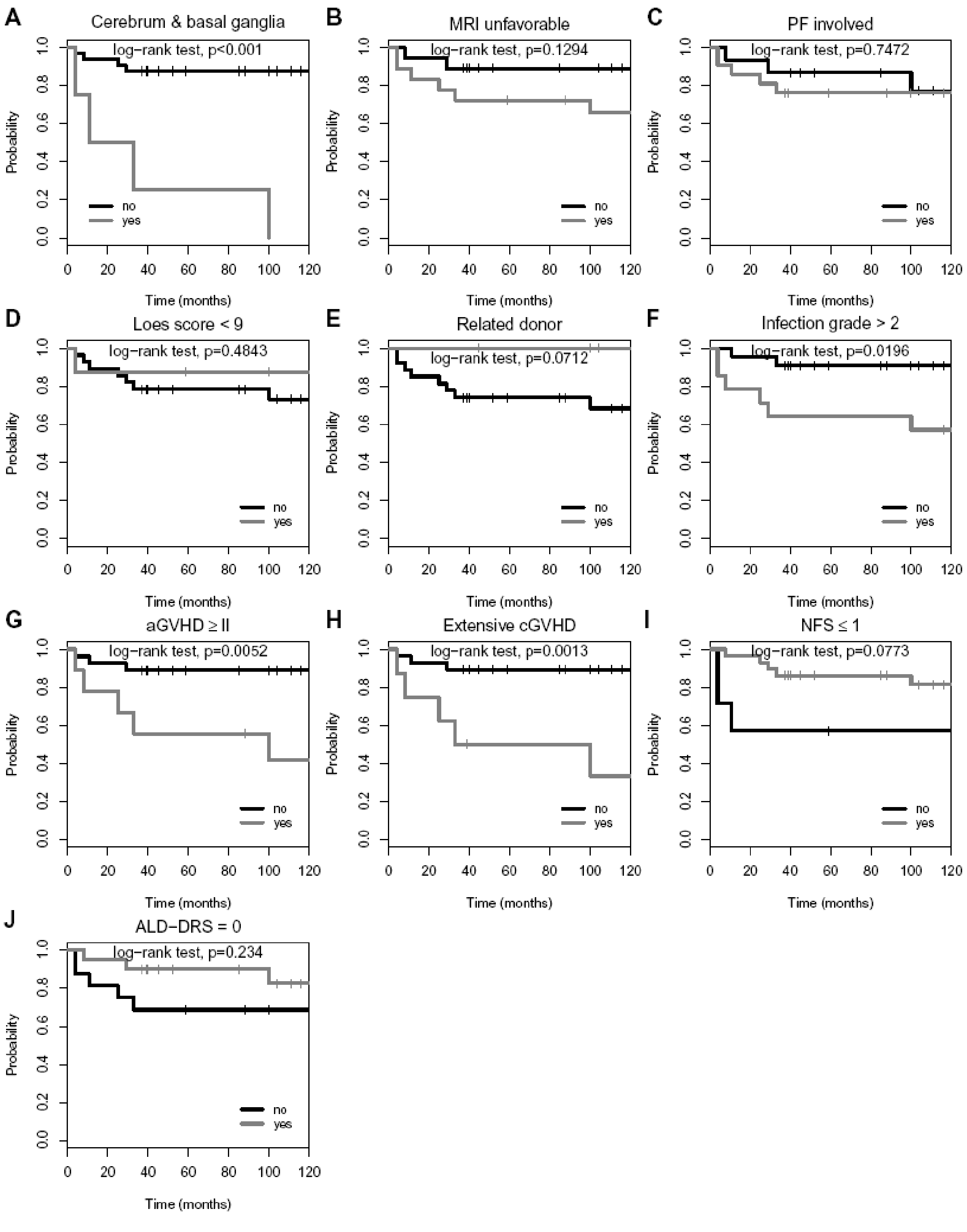

eFigure 2A. Probability of 10-Year Overall Survival

Patients were stratified by respective covariates as indicated. Parameter present: grey line; parameter absent: black line. Dashes indicate censored patients.

© 2018 Kühl J-S et al. *JAMA Network Open.*

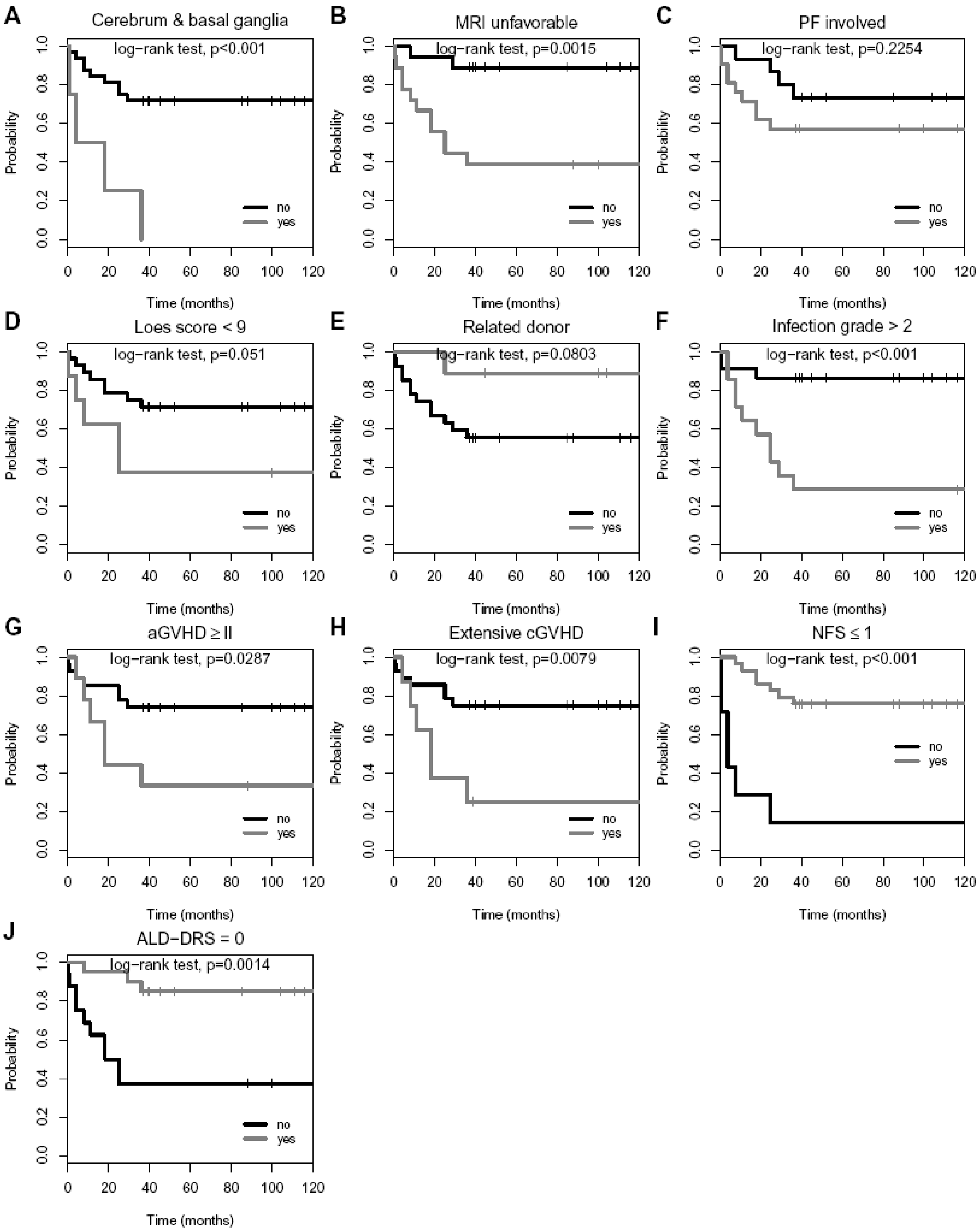

eFigure 2B. Probability of Survival Without Major Functional Disabilities
Patients were stratified by respective covariates as indicated. Parameter present: grey line; parameter absent: black line. Dashes indicate censored patients.

© 2018 Kühl J-S et al. *JAMA Network Open.*

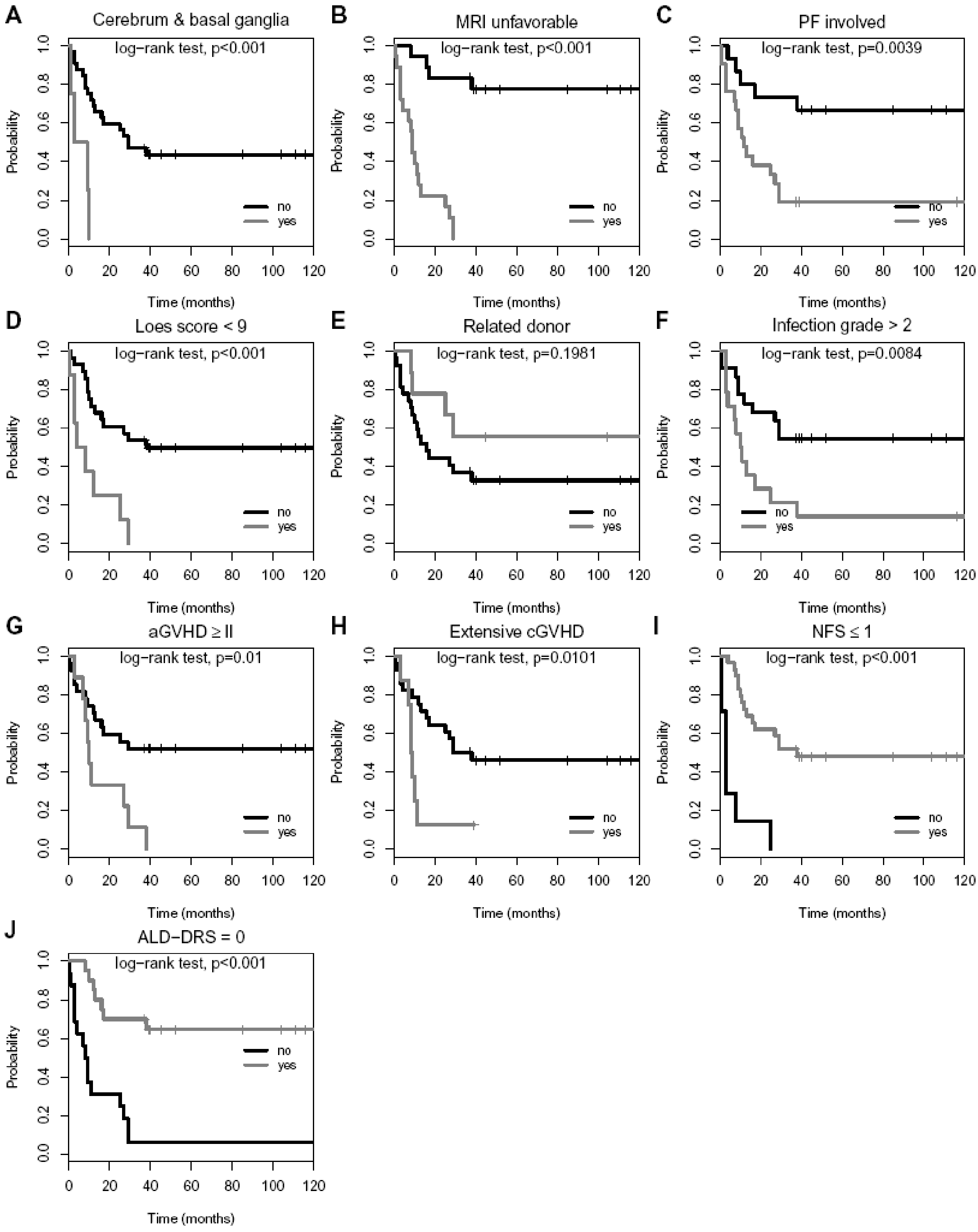

eFigure 2C. Probability of Event-Free Survival (i.e. Survival Without Gain in Disability Level)

Patients were stratified by respective covariates as indicated. Parameter present: grey line; parameter absent: black line. Dashes indicate censored patients.

© 2018 Kühl J-S et al. *JAMA Network Open.*

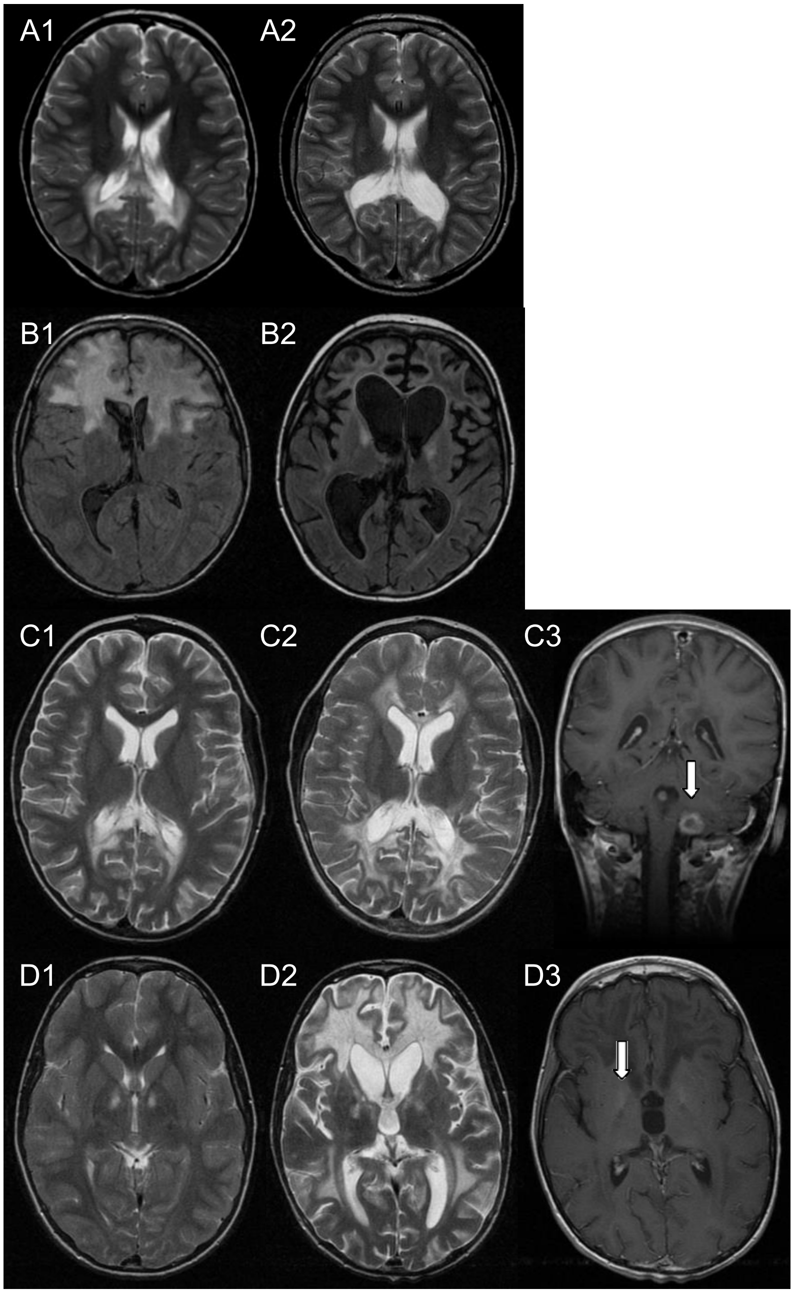

eFigure 3. Favorable (A) and Unfavorable (B-D) Neuroimages
A: T2-weighted axial magnetic resonance images of classical paritooccipital pattern before (A1; Loes score (LS) = 8.0) and 89 months post-transplant (A2; LS = 10). Only local atrophy, stable neurocognition. **B**: FLAIR images of frontal pattern before (B1; LS = 6.5) and 14 months post-transplant (B2; LS = 16.5). Severe global atrophy with frontal predominance. Death from disease progression after 25 months. **C**: T2-weighted images of a pattern with cerebellar involvement: in comparison to 2 months post-transplant (C1; LS = 8.5), at 22 months (C2; LS = 22) both paritooccipital and frontal new white matter lesions. The coronar T1 image (C3) reveals persistent cerebellar Gadolinium uptake 8 months post-transplant (arrow). Progression and death 100 months post-transplant. **D**: T2-weighted images of a pattern affecting the anterior thalamus and projection fibers before (D1; LS = 2) and 19 months post-transplant (D2; LS = 17). Severe increment of white matter demyelination is observed. The T1 image (D3) of the latter examination demonstrates sustained Gadolinium uptake (arrow). Dramatic clinical deterioration and death after 33 months.

© 2018 Kühl J-S et al. *JAMA Network Open.*

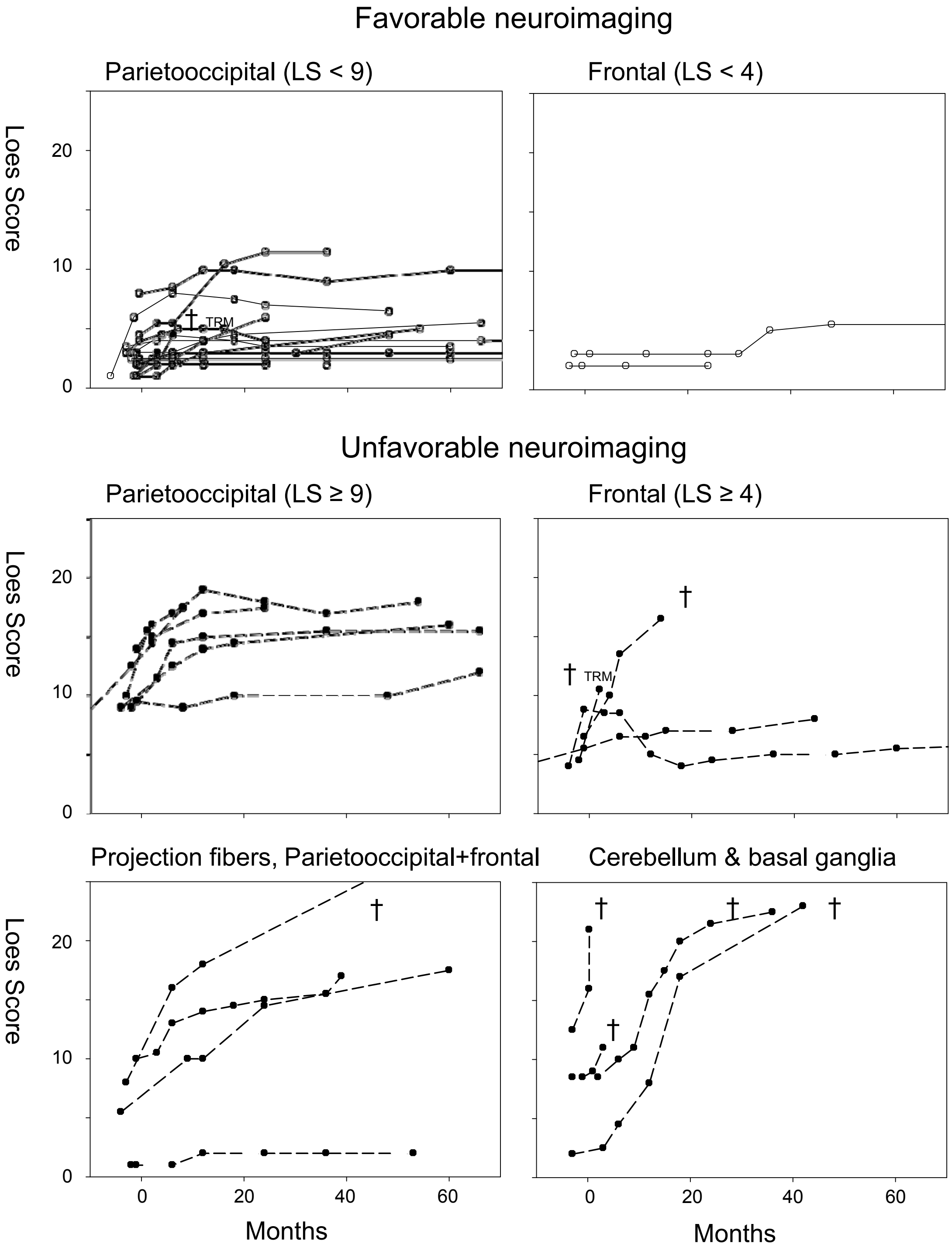

eFigure 4. Demyelinating Lesions in Neuroimaging

Illustrated are the individual Loes scores (LS) of all 36 patients at various time points. Patients are separated for different MRI patterns and the extent of demyelination. The Loes score ranges from 0 - 34 points and increases with the number of demyelinating lesions. Therapy related deaths († TRM; 2 patients with parietooccipital pattern and LS < 9, 1 patient with frontal pattern and LS ≥ 4) as well as deaths from disease progression are indicated.

© 2018 Kühl J-S et al. *JAMA Network Open.*

eTable. Overview on Retrospective Transplant Studies and Prospective Gene Therapy Study for CCALD

Study	All/early pts. (*N*=)	Transplantation	Conditioning	Graft failure	Acute GVHD	TRM	OS	Neuro(psycho)logical stable survival
Peters C et al., 2004<br>Multi-center	94<br>NFS=0: 32<br>ALD-DRS=0: 13	Matched related 33<br>Matched unrelated 31<br>BM 82, CB 12	BuCy 47<br>TBI-based 46	13/80 (14 %)	12 %<br>(Grade III-IV)	14 %	56 % @8 yr	ΔNFS=0: 18/32 (56 %)<br>ΔALD-DRS=0:<br>7/13 (54 %)
Beam D et al., 2007<br>Single center	12<br>LS<10: 6	Unrelated cord blood	BuCy only	1	2<br>(Grade III-IV)	2	67% @3 mo	5 (42 %)
Miller W et al., 2011<br>Single center	60<br>NFS=0: 23<br>LS<10: 30	(Un)related BM (10)18<br>Unrelated CB 32<br>Matched 27	BuCy 28<br>TBICy 16<br>RIC 16	3<br>(deceased only)	18 %<br>(Grade II-IV)	8 % @d+100	47/ 60<br>75 % @5 yr	ΔNFS=0: >75%
Kato S et al., 2016<br>Registry report *	84<br>including adults	(Un)related BM *<br>Unrelated CB	BuCy*<br>RIC	18 %	NR	NR	79 %	NR
Mitchell R et al., 2013<br>Registry report **	15<br>Status NR	NR**<br>[BM 47 %]	BuCy	1	NR**<br>[III-IV: 14 %]	NR**<br>[19 %@1 yr]	73 % @5 yr	NR
Van den Broek B et al., 2018<br>Multi-center ***	56<br>no neuropathy: 31	Unrelated cord blood	BuCy (83%)	NR***<br>[12 %]	NR***<br>[III-IV: 20 %]	NR***<br>[25 %]	35/56 @6 yr<br>(63 %)	Stable neuropathy:<br>24/48 (50 %)
Fernandes J et al., 2018<br>Two centers	9<br>Loes <10: 4	Haploidentical	RIC only	4	2<br>(Grade III-IV)	1	8/9<br>FU 29 mo.	NFS=0: 3/9<br>(one pt. with LS=21)
Kühl J et al.<br>Single center	36<br>NFS=0: 21<br>ALD-DRS=0: 20	Matched related 9<br>Matched unrelated 27<br>BM 26, PBSC 9, CB 1	Bu/Cy only	0	3 (8 %)<br>(Grade III-IV)	3 (8 %)	27/36<br>81 % @8 yr	ΔNFS=0: 17/21 (81 %)<br>ΔALD-DRS=0:<br>13/20 (65 %)
Eichler F et al., 2017<br>Multi-center	17<br>All NFS=0	Autologous PBSC<br>(Lentivirus-transfected)	BuCy only	0	0	0	15/17<br>FU 29 mo.	ΔNFS=0: 12/17 (71 %)

Early pts.: Patients with early/less advanced disease as indicated (NFS=0: normal neurological function score; ALD-DRS=0: no ALD-related disability: LS<10: Loes score < 10 points (possible range 0 – 34, maximum indicates worst status)). Transplantation: BM: bone marrow; CB: cord blood; PBSC: peripheral blood stem cells. Conditioning: BuCy: busulfan/cyclophosphamide; TBI: total body irradiation; RIC: reduced intensity conditioning. GVHD: graft-versus-host disease. TRM: transplant-related mortality at indicated time (days/years). OS: overall survival at indicated time (months/years); FU: follow-up. ΔNFS=0/ ΔALD-DRS=0: no gain in deficits/disabilities for those patients without deficits/disabilities prior to treatment. *Kato S et al., NR not reported, specific numbers not reported. **Mitchell R et al.: specific numbers not reported (in brackets numbers for entire cohort of 53 patients with inherited metabolic diseases. ***van den Broek B et al.: specific numbers not reported (in brackets numbers for entire cohort of 169 patients with inherited leukodystrophies.

© 2018 Kühl J-S et al. *JAMA Network Open.*
